# Cell identity and 5-hydroxymethylcytosine

**DOI:** 10.1186/s13072-025-00601-w

**Published:** 2025-06-19

**Authors:** Floris Honig, Adele Murrell

**Affiliations:** Department of Life Sciences, University of Bath, Claverton Down, Bath, BA2 7AY UK

**Keywords:** 5-hydroxymethylcytosine, Cell identity, Cell conversions, Epigenetic mechanisms, Epigenetic barriers

## Abstract

Epigenetic factors underlie cellular identity through the regulation of transcriptional networks that establish a cell’s phenotype and function. Cell conversions are directed by transcription factor binding at target DNA which induce changes to identity-specific gene regulatory programs. The degree of cell plasticity is determined by the interplay of epigenetic mechanisms to create a landscape susceptible to such binding events. 5-hydroxymethylcytosine, a key intermediate during the process of DNA demethylation, is an epigenetic modification involved in controlling these epigenetic dynamics related to cell identity. Here, the role of 5-hydroxcymethylcytosine during cell identity conversions, including its relationship with other main epigenetic mechanisms, is reviewed.

## Introduction

Gene expression underlies cell identity through the conversion of genetic information stored within DNA sequences into gene products that ultimately constitute a cell’s function [1, 2]. The relative amount and type of such biochemical material produced by a cell, including RNA and protein, distinguishes one cell from another [1, 2]. Every individual cell expresses thousands of genes concurrently in response to intra- and extracellular stimuli to establish its cell type-specific phenotype and function [3]. These interactions between genes and regulators form complex signalling pathways also known as gene regulatory networks (GRNs) and play an important role in cell identity control [4, 5]. For example, GRNs determine the main regulatory steps required during developmental processes such as organogenesis and lineage specification [6, 7]. Moreover, reconfiguration of GRNs allows for rapid cell identity conversion of one cell type to another [8–10].

The induction and maintenance of these gene expression programs is controlled by transcription factor binding at gene regulatory DNA regions of target genes [11, 12]. Such binding events facilitate the recruitment of other transcription factors, cofactors and transcriptional machinery to regulate gene expression [12]. The ability of transcription factors to engage with their target DNA is highly dependent on a cell’s chromatin and nuclear architecture, which is modulated by distinct epigenetic mechanisms, including histone modifications and DNA (de)methylation [13–15]. The interplay between these modifications creates a dynamic landscape that can be either permissive or repressive to transcriptional changes [16, 17]. Overcoming these epigenetic barriers is fundamental to cell identity conversion.

In this review, the role of the epigenetic mark 5-hydroxymethylcytosine (5hmC) in cell identity control is elucidated, with a particular focus on cellular conversions.

### 5hmC: A key DNA demethylation intermediate and stable epigenetic mark

**Fig. 1:**
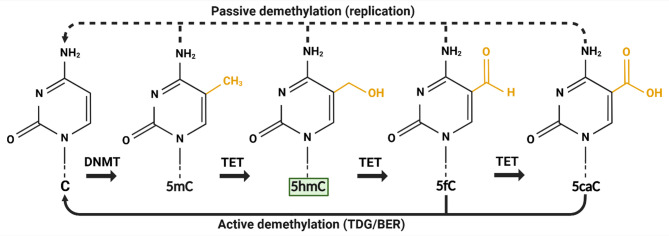
5hmC as an intermediate during passive and active DNA demethylation of cytosine modifications. Cytosine is methylated to 5-methylcytosine by DNA methyltransferases. Ten-eleven translocation enzymes convert 5-methylcytosine into 5-hydroxymethylcytosine (highlighted in green), and subsequently into 5-formylcytosine and 5-carboxylcytosine via oxidation reactions in a stepwise manner. Passive demethylation of all cytosine modifications happens during DNA replication through DNA methyltransferases dysfunction or suppression. Active demethylation occurs via base excision repair following excision of 5-formylcytosine or 5-carboxylcytosine by thymine glycosylases. Created in BioRender: https://BioRender.com/w40a603

Although 5hmC was originally detected in viral DNA in the 1950s [18] and in mammalian DNA in 1972 [19], it was not until 2009 that its novel function in epigenetic regulation via DNA demethylation was recognised [20, 21]. Since then, its role as key intermediate in the removal of repressive epigenetic mark 5-methylcytosine (5mC; which is deposited by DNA methyltransferases/DNMTs)^22^ via both passive and active DNA demethylation has been widely demonstrated (Fig. 1). Ten-eleven translocation (TET) enzymes oxidise 5mC into 5hmC, before further stepwise generation of 5-formylcytosine (5fC) and 5-carboxylcytosine (5caC) [23]. The latter two cytosine derivates can be recognised by thymine DNA glycosylases (TDG). 5fC and 5caC are then subjected to base excision repair (BER) to form unmodified cytosine (C) via active demethylation [24]. Passive demethylation can also occur during DNA replication in the absence of DNMTs [22]. It was later discovered that 5hmC may also act as a stable epigenetic modification suggesting that it may direct biological processes independently of its intermediate function for DNA demethylation [25]. Earlier it was shown that various tissues and cell types contain differing amount of global 5hmC suggesting that accumulation and distribution of 5hmC may be cell type specific [26, 27]. Altogether, 5hmC may maintain an epigenetic role linked to cell identity as essential intermediate during DNA demethylation and possibly as stable epigenetic mark.

### Cellular conversions and the epigenetic landscape

Cellular conversions, also known as cell fate conversions, involve the transition of one cell identity to another and can occur via several distinct routes: cell differentiation, somatic cell reprogramming, and transdifferentiation [28–30]. Cell differentiation is the process through which cells become a more specialised, differentiated cell identity. This often occurs via a stepwise progression from (pluripotent) stem cells into precursor cells followed by mature cells. Conversely, somatic cell reprogramming, or dedifferentiation, involves the reversion to a less differentiated state and primarily revolves around the reprogramming of somatic cells to a pluripotent state. Lastly, transdifferentiation, or direct reprogramming, encompasses the direct switch between two cell identities without going through an intermediate state.

**Fig. 2:**
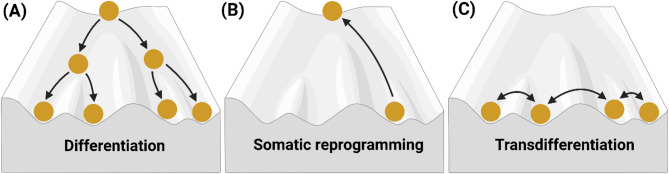
Cell conversion routes displayed on Waddington’s epigenetic landscape. **(A)** The several trajectories through the valleys of Waddington’s landscape encompass the stepwise progression of (pluripotent) stem cells to distinct specialised, mature cells. **(B)** The scaling up the hill represents the reversion of a cell to a less differentiated identity known as somatic cell reprogramming. **(C)** Transdifferentiation (or direct reprogramming) is illustrated by trajectories across ridges between specialised cell identities. Adapted from Waddington’s original model [31]. Created in BioRender: https://BioRender.com/n40n501

The above-mentioned cell conversion routes can be described in the context of the epigenetic landscape (Fig. 2). Waddington’s traditional model of cell differentiation postulated in 1957 describes the stepwise specification of pluripotent stem cells into their mature differentiated state as a unidirectional, irreversible process (Fig. 2A) [31]. This is illustrated by using a marble (representing a cell) rolling down a hill into different grooves (cell fates) as a metaphor [31]. The marble’s trajectory determines the cell’s final, differentiated identity and the ridges represent the epigenetic barriers (e.g. DNA methylation and chromatin modifications [15–17]) that prevent differentiated cells from switching identity. However, with the emergence of direct cell conversion technologies able to overcome some of these barriers [28–30, 32]Waddington’s original model has since been adapted to include somatic cell reprogramming (Fig. 2B) and transdifferentiation (Fig. 2C) trajectories.

### 5hmC and cellular conversions

In the following sections, the role of TET-mediated DNA hydroxymethylation during cellular conversions is elucidated.

### 5hmC and somatic cell reprogramming

Several studies have shown the importance of TET-mediated DNA demethylation in somatic cell reprogramming to pluripotency [33–38]. Gao et al. revealed that total 5hmC is increased during the reprogramming of mouse embryonic fibroblasts (MEFs) to induced pluripotent stem cells (iPSCs) using the OCT4, SOX2, KLF4 and c-MYC (OSKM) Yamanaka cocktail [33]. This 5hmC enrichment was mainly observed at genomic regions known to be involved in regulating pluripotency. Specifically, the crucial function of TET1 and 5hmC in demethylating the promoter and enhancer regions of *Oct4* throughout the early stages of reprogramming was highlighted. Moreover, TET1 could replace OCT4 to generate fully pluripotent iPSCs. Similar observations were made during a functional screen of 29 epigenetic factors using the same OSKM somatic cell reprogramming system [34]. TET2 was found to be recruited to the *Nanog* and *Esrrb* pluripotency loci upon reprogramming, which coincided with a significant increase of 5hmC at these loci. Additionally, knockdown of TET2 in MEFs prevented such elevated levels of 5hmC. Consistent findings were observed during C/EBPα-enhanced reprogramming of B cells into iPSCs [35]. Here, a TET2-dependent gain in 5hmC was detected at gene regulatory elements of key pluripotency factors *Nanog*, *Oct4* and *Klf4*. The same authors also confirmed these results during the reprogramming of fibroblasts to iPSCs [35].

Elsewhere, the combined significance of TET1- and TET2-mediated DNA hydroxymethylation during iPSC induction of fibroblasts has been shown [36]. Reprogrammed iPSCs displayed increased *Tet1* and *Tet2* mRNA levels, as well as 5hmC content, when compared to the original fibroblast population. These acquired levels were identical to what is typically observed in mouse embryonic stem cells (mESCs). Similarly, when *Tet1* mRNA is depleted from mESCs using RNA interference methods, a significant decrease in 5hmC and a loss of stem cell identity is observed [37]. Wang et al. obtained comparable results in a human iPSC reprogramming model [38]. 5hmC content and *TET1* mRNA levels were shown to significantly increase during the reprogramming of human fibroblasts to iPSCs. Short hairpin RNA-mediated *TET1* silencing was shown to reduce 5hmC levels and decrease the number of alkaline phosphatase-positive iPSC colonies, which is a marker of pluripotency in iPSCs. When the authors performed a genome-wide DNA hydroxymethylation comparison of human iPSCs of distinct origins to human ESCs, 5hmC patterns were generally identical. However, iPSC showed more epigenetic variation, as evidenced by the presence of several large-scale abnormal hydroxymethylation hotspots in subtelomeric regions in iPSCs that were not detected in ESCs.

A few studies have focused on the individual roles of each Tet enzyme (Tet1, Tet2 and Tet3) and their importance in mediating active demethylation during somatic reprogramming to pluripotency [39, 40]. In a Tet knockout model of MEFs it was shown that Tet2 knockout reduced reprogramming efficiency by 70% and a total knockout of all Tet enzymes entirely blocked reprogramming [39]. Contrarily, Tet1 knockout resulted in a slight increased number of alkaline phosphatase-positive colonies, whereas Tet3 deletion had a negligible effect. The inability of Tet1-3 knockout MEFs to reprogram to iPSCs was demonstrated to be TDG-dependent and shown to be halted at the mesenchymal-to-epithelial transition. Another study confirmed these results by underlining the significance of further 5hmC oxidation to 5fC and 5caC during the reprogramming of MEFs to iPSCs [40]. Specifically, somatic reprogramming to iPSCs of Tet2-deficient MEFs could only be rescued via the re-expression of Tet dioxygenases able to oxidise to 5fC and 5caC.

Taken together, these studies highlight the crucial role of (active) TET-mediated DNA hydroxymethylation in reactivating pluripotency during somatic cell reprogramming to iPSCs.

### 5hmC and cell differentiation

5hmC dynamics are also important for directing the fate of cells arising from the three primary germ layers: the ectoderm, mesoderm, and endoderm.

The high abundance of 5hmC in the mammalian brain points to its potential relevance for neuronal development and function [20] and evidence of this importance of 5hmC dynamics during neurogenesis has been widely reported [41–48]. Hahn et al. discovered that global 5hmC levels accumulate with neuronal differentiation of the mouse brain in vivo, whereby double the amount of 5hmC was detected in isolated neurons when compared to neural progenitor cells [41]. Further genome-wide profiling revealed that 5hmC was enriched at the gene bodies of transcriptionally active neuronal markers. Intriguingly, inhibition of TET2 and TET3 resulted in neuronal differentiation defects. An increase in levels of 5hmC has also been observed in developing granule cells of mice, with the highest levels of 5hmC mapping to exon start sites of genes related to axon guidance and ion channels [42]. Knockdown of *Tet1* and *Tet3* using RNA interference in developing granule cells in an ex vivo system resulted in decreased 5hmC levels and downregulation of these same genes [42]. Similar results were obtained in the developing postmitotic Purkinje cells of mice in vivo [43, 44]. Zhou et al. detected global waves of methylation and hydroxymethylation specific to Purkinje cell maturation during normal development [43]. It was further shown that DNA methyltransferase 1 (DNMT1) and TET1 mirrored these patterns of 5mC and 5hmC, respectively. In another study, a lack of 5hmC accumulation, mediated by triple knockout of all three TET proteins, prevented normal transcriptional and epigenetic maturation of Purkinje cells [44]. Therefore, it was concluded that continuous accumulation and removal of 5hmC is necessary for the development of adult Purkinje cells. Likewise, during the development of the mouse main olfactory epithelium in vivo, mimicked by the stepwise differentiation of multipotent stem cells into neuronal progenitors followed by mature olfactory sensory neurons, gene body profiles of 5hmC correlated with gene expression at each cell stage [45].

The forward reprogramming of pluripotent stem cells into neurons provides further insights into the role of TET-mediated DNA hydroxymethylation during neurogenesis [46–48]. The terminal differentiation of mouse ESCs into neurons has been shown to be impaired upon TET3 knockout [46]. Furthermore, genome-wide 5hmC patterns are evidenced to be highly dynamic during the stepwise differentiation of human ESCs into neural precursors and dopamine neurons [47]. Throughout this process, 5hmC enrichment in gene bodies was shown to be associated with transcriptional activation at neurogenesis-specific genes, including *RGMA*, *AKT1* and *NOTCH1*. Similarly, forebrain organoids derived from human iPSCs showed differential hydroxymethylation at loci related to each specific developmental stage [48]. Dynamic changes of 5hmC may be essential to mammalian neuronal development. However, since TET proteins also have a number of non-catalytic functions, we cannot definitively distinguish between TET and 5hmC functions.

Remodelling of 5hmC has also been linked to hematopoietic stem cell lineage commitment. This has been demonstrated by alterations in 5hmC that were reportedly detected over the course of T cell differentiation [49–51]. Tsagaratou et al. showed that at the distinct stages of in vitro mouse T cell development in the thymus and periphery, 5hmC is enriched at the gene bodies and enhancers of highly transcribed genes [49]. Particularly, key regulatory genes associated with T cell development displayed high intragenic 5hmC levels in precursor cells. Similar results were reported in a human in vitro model of CD4^+^ T cell differentiation [50]. Here, 5hmC was primarily located at genic regions in these cell types and correlated with active gene transcription. The differentiation of naïve CD4^+^ cells into T helper cells resulted in a global loss of 5hmC. However, 5hmC enrichment did occur at genomic regions known to be linked to T cell differentiation, such as *CCR2* and *CCR5*. Nackauchi and colleagues further underlined that 5hmC patterns during human haematopoiesis are associated with active transcription and are enriched at critical hematopoietic regulators [51]. Additionally, *TET2* knockout caused disrupted megakaryocytic and erythroid differentiation of hematopoietic stem cells both in vitro and in vivo models. Overall, these studies show 5hmC-mediated DNA demethylation during haematopoiesis.

The importance of TET-mediated DNA demethylation via the conversion of 5mC to 5hmC in regulating skeletal muscle differentiation has also been explored [52–54]. Zhong et al. demonstrated the mechanism through which Tet2 mediates in vitro myogenic differentiation of murine C2C12 myoblast by demethylating promoter regions of key skeletal muscle genes, such as *Myog* [52]. These results are echoed by an investigation that has since underlined the role of Tet2 in supporting skeletal muscle regeneration by regulating the differentiation and fusion of primary mouse myoblast both in vivo and in vitro [53]. Again, Tet2-driven DNA demethylation of the enhancer region of *Myog*, including subsequent transcriptional activation, was found to be critical to myogenic differentiation of these cells. Increased DNA demethylation was also observed during myogenic differentiation of human myoblast obtained from muscle biopsies, which was linked to increased *TET1-2* mRNA and 5hmC levels [55]. Together, these results highlight the involvement of 5hmC in skeletal muscle differentiation of myoblasts.

Other cell types originating from the mesoderm have also been implicated with the acquisition of 5hmC during differentiation. In vitro chondrogenic differentiation of ATDC5 progenitor cells was demonstrated to be accompanied by increased 5hmC and *Tet1-3* mRNA levels [56]. Subsequent loss-of-function experiments, whereby *Tet1* knockdown resulted in reduced 5hmC levels and compromised chondrogenic differentiation, suggest an essential role for TET1-mediated hydroxymethylation during chondrogenesis. This was later confirmed in a follow-up study, which revealed the importance of TET1 in facilitating hydroxymethylation at target sites of master chondrogenic transcription factor SOX9 [57]. Yoo et al. discovered a similar mechanism of action during the in vitro adipogenic differentiation of murine 3T3-L1 preadipocytes [58]. *Tet1* and *Tet2* mRNA transcripts, as well as global 5hmC levels, were found to be upregulated upon adipogenesis. Specifically, 5hmC was enriched at the locus of the positive adipogenic regulator, peroxisome proliferator-activated receptor γ gene (*Pparg)*. Knockdown of *Tet1* and *Tet2* inhibited 5hmC accumulation at the *Pparg* locus and blocked adipogenesis. Another study that made use of the same cell model showed that the TET enzymes promoted hydroxymethylation at the enhancer regions of genes regulating adipogenesis [59].

The differentiation of cells from endodermal origin, including hepatocytes and pancreatic cells, has been associated with dynamic changes in hydroxymethylation [60–62]. TET1-mediated 5hmC enrichment at the promoter region of hepatic master regulator *HNF4A* is essential to switching on the hepatocyte transcriptional program during the in vitro differentiation of human HepaRG cells [60]. In this same cell model, it has been shown that global 5hmC increased after one week of differentiation [61]. Moreover, TET inhibition and changes to the metabolic environment impaired hepatocyte differentiation. This impairment has been linked to decreased 5hmC accumulation, including reduced 5hmC enrichment at the previously mentioned *HNF4A* promoter region. Dynamic changes to the hydroxymethylation landscape have been reported to drive the pancreatic in vitro differentiation of human ESCs [62]. An initial decrease in global 5hmC levels was observed during the ESC to definitive endoderm transition, which returned to near-initial levels upon further pancreatic differentiation. Additionally, the authors showed that 5hmC-marked genomic regions became demethylated in descendent lineages. Together, this work demonstrates the importance of the 5hmC landscape in driving endodermal differentiation to different cell types.

### 5hmC and transdifferentiation

A limited number of studies have investigated TET-mediated DNA hydroxymethylation in the context of transdifferentiation [63–65]. One in vitro study focused on the direct conversion of MEFs into functional neurons mediated by Tet3^64^. Specifically, it was shown that ectopic expression of Tet3 efficiently converted fibroblasts to functional neurons, expressing mature neuronal markers (Tuj1, Synapsin and NeuN), and electrophysiological functions. On the contrary, addition of Tet3 shRNA vastly inhibited the formation of these functional neurons. Moreover, global 5hmC levels steadily increased over the time course of transdifferentiation. Related to this, genome-wide 5hmC revealed that 5hmC was highly enriched in neuron-specific genes in the converted neurons and correlated with gene expression at these loci. A related mechanism of action has been demonstrated using the direct in vitro reprogramming of mouse fibroblasts to neurons via Ascl1 induction [64]. Here, the authors suggested that the expression of neuronal developmentally upregulated genes was associated with 5hmC acquisition. Furthermore, de novo methylation by DNA methyltransferase 3 alpha (DNMT3A) was found to be essential to successful transdifferentiation in this cell model by depositing 5mC substrate at developmental genes for subsequent hydroxymethylation to initiate gene activation. CEBPα-induced transdifferentiation of pre-B cells into macrophages is another in vitro cell system shown to be facilitated by TET-mediated hydroxymethylation [65]. Kallin et al. found that CEBPα binds to regulatory regions of Tet2, activating its expression. Knockdown of Tet2 was demonstrated to result in reduced expression of myeloid target genes and thus impaired the transdifferentiation of pre-B cells into macrophages. Furthermore, it was demonstrated that Tet2-mediated activation of myeloid target genes was accompanied by promoter hydroxymethylation at these genes. This evidence suggests that TET-mediated DNA hydroxymethylation is associated with transdifferentiation models.

**Fig. 3:**
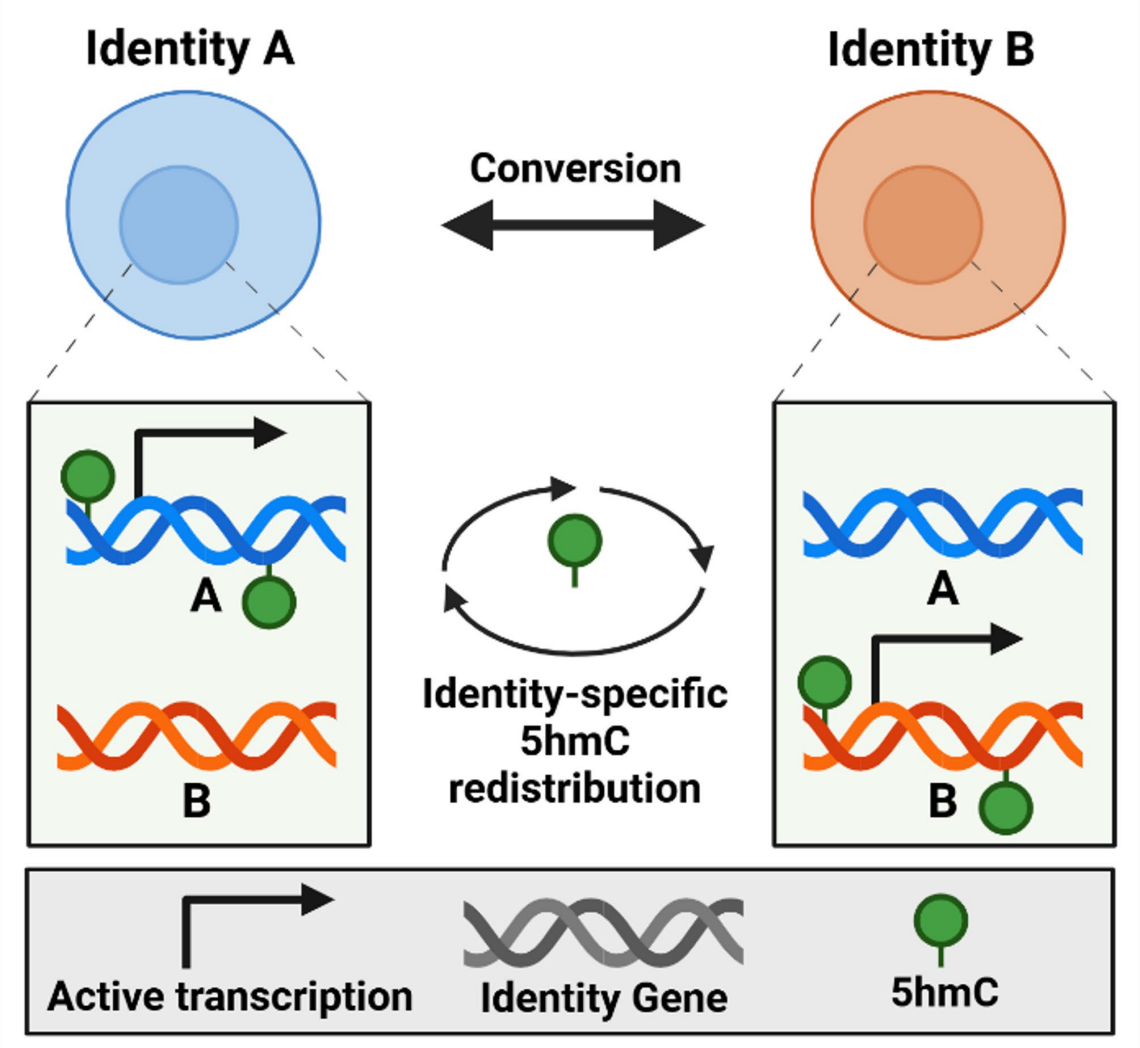
5hmC dynamics during cell conversions. 5hmC remodelling takes place at identity genes and associated regulatory regions during cell conversions, thereby activating identity-specific gene expression. Created in BioRender: https://BioRender.com/r59v578

In summary, 5hmC dynamics play an important role during cell conversions through regulating cell identity-specific gene expression (Fig. 3). Somatic cell reprogramming to iPSCs is linked to increased levels of global 5hmC and enrichment of 5hmC at gene regulatory regions known to be involved in pluripotency. Moreover, TET1 and TET2 knockdown decrease reprogramming efficiency, where TET1 can replace OCT4 during OSKM-induced reprogramming. During cell differentiation into the three germ layers both the global levels and distribution of 5hmC are evidently highly dynamic. Specifically, TET-mediated enrichment of 5hmC at loci of key developmental genes takes place, which correlates with cell-type specific gene expression. Finally, similar mechanisms appear to be at play during transdifferentiation, though this has been studied to a much lesser extent than somatic cell reprogramming and cell differentiation. Regulatory regions of key genes of target cell identities have been shown to become hydroxymethylated and inhibition of TET enzymes appear to impair transdifferentiation.

### 5fC, 5caC and cell identity

A similar role for 5fC has been described as that of 5hmC in the context of cell identity [66–68]. For example, in vivo genome-wide profiling of 5fC in mouse tissue revealed tissue-specific 5fC profiles at active developmental enhancers [66]. Related, the genome-wide distribution of 5fC in mouse ESCs showed enrichment at CpG islands of promoters of active genes and 5fC was relatively more enriched than 5mC and 5hmC [67]. TDG knockdown in these cells resulted in an increase of 5fC at CpG islands near genes associated with cell morphogenesis and differentiation, suggesting a possible role for 5fC in differentiation [67]. Another study revealed that during human early embryonic development 5fC is highly dynamic and marked promoters of active genes relevant to each developmental stage [68]. Additional in vitro studies profiling global 5fC levels further underline the relevance of 5fC during cell conversions [69–71]. For instance, Mulholland et al. described TET2-dependent 5fC formation during the naive to primed pluripotency transition in mouse ESCs [69]. On the contrary, a global decrease in 5fC formation was observed during the differentiation of mouse ESCs into neurons [70]. A similar decrease in 5fC levels was seen upon differentiation of neural stem cells isolated from the brain of adult mice [70]. Overall, 5fC shows similar features in cell identity control as those described for 5hmC.

A limited number of studies have focused on the role of 5caC during cell conversions [72, 73]. Wheldon et al. showed that 5caC levels accumulated during in vitro neuronal and glial differentiation of mouse neural stem cells [72]. 5caC was enriched at cell-type specific promoters during differentiation and TDG knockdown led to increased 5caC levels in differentiating cells. Similar observations were made by the same authors in an in vitro differentiation model of human iPSCs to hepatic-like cells [73]. Namely, 5caC accumulated globally and showed specific enrichment at promoter regions of genes involved in hepatic specification. Further independent studies are necessary to unravel the exact role of 5caC in cell identity control.

### 5hmC and epigenetic mechanisms

As discussed, the epigenetic landscape is often described as the collection of site-specific chromatin modifications, including DNA (de)methylation, histone modifications and the binding of structural proteins [13–15]. The interplay among these epigenetic mechanisms, including 5hmC, establishes chromatin states that are either permissive or repressive for transcription and thus underlies cell identity conversions [16, 74, 75]. In the following section, the relationship between 5hmC and epigenetic mechanisms during cell conversions is described, focusing on histone modifications, chromatin remodelling and transcription factor binding.

5hmC has been shown to be associated with histone modifications in human embryonic stem cells (hESCs) [76, 77]. For example, 5hmC was enriched at regions marked by H3K4me1 and H3K27ac and associated with active enhancers [76]. These findings are further supported by another study showing positive correlations between 5hmC and histone marks H3K4me1-3 and H3K27ac in hESCs [77]. Conversely, lower correlation levels were seen for other histone marks, including H3K9ac, H3K9me3 and H3K36me3, suggesting that 5hmC is primarily associated with more accessible chromatin in hESCs. This is in accordance with evidence of reduced levels of global 5hmC and a loss of stem cell identity following knockdown of *Tet1* in mESC, which was accompanied by increased H3K9me3 levels [37]. TET-mediated DNA hydroxymethylation is also essential for the establishment and maintenance of bivalent domains (H3K4me3 and H3K27me3) that are poised for transcription, mainly at development genes in mESCs [78]. Others showed that polycomb repressive complex 2 recruits Tet1 and 5hmC at such bivalent promoters [79]. Similar interactions between TET-mediated hydroxymethylation and active chromatin modifications associated with gene expression were observed during somatic reprogramming to pluripotency [33–35]. Pluripotency loci marked by 5hmC showed enrichment in H3K4me2 and H3K4me3 histone marks in reprogrammed iPSCs in these conversion systems [33–35]and a decrease in repressive histone mark H3K27me3 occupancy [33, 34]. Together, these results indicate that 5hmC associates with active histone modifications to regulate pluripotency in ESCs and during somatic reprogramming.

Associations between 5hmC and histone modifications have also been reported during cell differentiation. Neurogenesis investigations have linked 5hmC occupancy at enhancer regions with enrichment of active or poised histone marks to promote active gene expression [41, 48]. Enhancers that gained 5hmC during the differentiation of human iPSCs to forebrain organoids were enriched with histone marks H3K4me1 and H3K4me3^49^. Similarly, a gain of intragenic 5hmC was associated with a gain of H3K4me3 at promoter regions during neuronal differentiation in mice, while a loss of H3K27me3 was correlated to increased gene body 5hmC levels [41]. A similar pattern is seen during human haematopoiesis [80, 81]. 5hmC enrichment at genic regions of highly expressed genes in differentiated blood cell types has been associated with H3K4me1, H3K4me3 and H3K27ac [81]. Conversely, an inverse trend between 5hmC levels and repressive marks H3K27me3 and H3K9me3 was observed [81]. Likewise, TET-mediated regions during the transition of pro-B cells to pre-B cells in B cell development were enriched for H3K4me1 and H3K4me3^81^. Furthermore, 5hmC showed a positive correlation with H3K4me1 and H3K27ac, whereas a lower correlation was seen for H3K4me3 and H3K27me3, during all stages of pancreatic differentiation [62]. Lastly, 5hmC, H3K4me1-3 and H3K27ac levels were enriched at regions regulating adipogenic differentiation [59]. Taken together, this evidence demonstrates that 5hmC is associated with poised and active histone marks, including H3K4me1-3 and H3K27ac, during cell differentiation to distinct cell types. By contrast, an opposite relationship is seen between 5hmC and repressive marks, such as H3K27me3 and H3K9me3. These investigations highlight the presence of 5hmC in the epigenetic landscape of genes that establish cellular differentiation. Such cell-specific 5hmC profiles are thus markers of cell identity.

Another key epigenetic mechanism that is relevant during cell conversions is the interplay between 5hmC and chromatin. Assay for Transposase-Accessible Chromatin (ATAC) sequencing profiles obtained during hematopoietic differentiation revealed that 5hmC enrichment correlated to both chromatin accessibility and gene expression at key hematopoietic regulators [51]. Strikingly, 5hmC was primarily enriched at the 5’ and 3’ ends of open chromatin sites. In line with this, regions that showed high 5hmC and increased chromatin accessibility during pancreatic differentiation, were found to be linked to cell stage-specific gene expression [62]. Another study profiling neural progenitor cell differentiation showed dynamic changes in 5hmC levels and chromatin accessibility linked to lineage-specific gene expression [82]. Gain and loss of 5hmC were closely related to open and closed regions of chromatin, respectively. Moreover, 5hmC levels increased prior to chromatin accessibility changes, mainly during the early phases of differentiation [82]. These results suggest that 5hmC generation may be relevant to pre-empt accessibility changes to direct cell identity-specific gene expression.

Considering this, studies have since begun investigating the relationship between 5hmC, chromatin accessibility and transcription factor binding during differentiation [80, 83, 84]. For instance, hydroxymethylation dynamics have been linked to heart development in both human and mice [83]. Deletion of Tet2/3 in a mouse model resulted in decreased expression of genes involved in cardiac development, which was linked to a reduction of 5hmC and chromatin accessibility [83]. Moreover, this 5hmC reduction was linked to compromised transcription factor binding of Ying Yang 1 (YY1; a key regulator of early heart development) and impaired chromatin organisation at genes important for heart development. A similar course of action was discovered during osteogenesis [84]. Namely, TET-dependent hydroxymethylation increased chromatin accessibility at osteogenic genes by facilitating the binding of osteoblast-specific transcription factor RUNX2. Moreover, RUNX2 directly interacted with TET enzymes at its binding regions to regulate DNA (de)methylation. Such interdependency between TET-mediated hydroxymethylation, chromatin accessibility, and transcription factor binding were also seen during pro-B cell differentiation [80]. In this case, binding of transcription factors E2A and PU.1, two key regulators of pro-B cells, was linked to TET activity and chromatin accessibility.

The connection between 5hmC, histone modifications, and transcription factor binding has been the focus of many investigations. Freudenberg. et al. discovered that *Tet1* depletion in mESCs prevented binding of signal transducer and activator of transcription 3 (Stat3) at target chromatin to regulate stem cell identity, which was linked to an increase in H3K9me3 at these binding sites [37]. In accordance with this, binding of Tfcp2l1 during somatic reprogramming was linked to Tet2-driven demethylation of regulatory pluripotency regions [35]. Moreover, an increase of 5hmC, chromatin accessibility, and histone marks H3K4me2 and H3K27ac, was observed at such regions. The binding of GATA2, RUNX1, FLI1 and ERG, which are hematopoietic transcription factors, were increased at sites marked by H3K4me1, H3K4me3 and H3K27ac in blood progenitor cells [81]. Particularly, binding of FLI1 and RUNX1 was further increased at sites that were further marked by 5hmC. Developmental transcription factor binding at enriched 5hmC regions was also observed during human foetal organogenesis [85]. Here, organ- and stage-specific transcription factor binding was observed at differentially hydroxymethylated regions, specifically in Alu elements, which are known to be involved in directing tissue-specific gene expression [86]. This same study further showed that 5hmC facilitated the binding of transcription factor TCF4 to E-box motifs in a protein binding microarray [86]. These studies highlight the ability of 5hmC to mediate transcription factor binding.

Lastly, the link between 5hmC and histone modifiers to regulate cell conversions has been a topic of interest. Histone deacetylase SIRT6 has been shown to regulate embryonic differentiation by controlling levels of H3K9ac and H3K56ac at the promoters of key pluripotency genes *Oct4*,* Sox2 and Nanog* [87]. Specifically, SIRT6 deletion in mouse ESCs resulted in a lack of inhibition of key pluripotency genes during embryonic differentiation and consequently skewed differentiation towards the neuroectoderm. TET expression and 5hmC was increased in SIRT6 knockout cells, which coincided with increased recruitment of OCT4 and SOX2 to *Tet1-*2 genes. Interestingly, short hairpin RNA-mediated depletion of *Tet1* or *Tet2* rescued the abnormal differentiation phenotype observed in SIRT6 knockout cells. This supports a potential role of SIRT6 in indirectly regulating TET levels to ensure normal differentiation. In another study, knockout of H3K9me2 demethylase Kdm3b prevented somatic reprogramming of MEFs to iPSCs [88]. Wild type cells showed reduced *Tet1* mRNA expression compared to their Kdm3b knockout counterparts after ten days of reprogramming. Intriguingly, simultaneous overexpression of Tet1 and Nanog in the Kdm3b knockout cells improved reprogramming efficiency. It was further demonstrated that genes that showed significant changes in both mRNA expression and 5hmC enrichment between wild type and Kdm3b knockout cells were associated with pluripotency and embryogenesis. These findings point at a possible connection between Kdm3b and Tet1. Lastly, in a primary human in vitro model the terminal differentiation of monocytes into macrophages or osteoclasts was impaired upon TET2 knockdown [89].Moreover, it was shown that TET2 knockdown hindered the binding of H3K4 methyltransferase SETD1A to myeloid-specific genes during differentiation. These observations support a possible indirect link between TET2 and enzymes histone modifier SETD1A during terminal differentiation of myeloid cells.

**Fig. 4:**
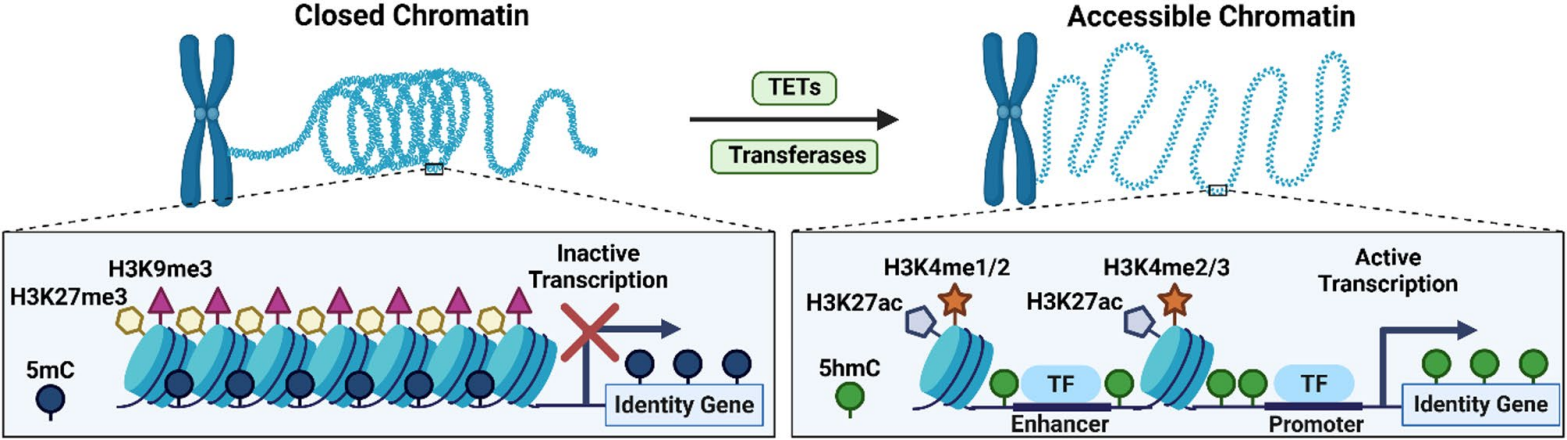
The interplay between 5hmC and epigenetic mechanisms modulate chromatin states to regulate identity-specific gene expression. Closed chromatin is associated with repressive epigenetic marks, including 5mC, H3K27me3 and H3K9me3, and is transcriptionally inactive. 5hmC comes together with other epigenetic marks, including H3K27ac and H3K4me1-3, at gene regulatory regions to establish chromatin permissive for transcription factor binding and active gene transcription. This transition between closed and accessible chromatin is mediated by TET enzymes and histone transferases. Created in BioRender: https://BioRender.com/w40a603

In sum, TET-mediated hydroxymethylation has been shown to underly cell conversions by cooperating with other epigenetic modifications to establish transcriptionally active open chromatin (Fig. 4).

### 5hmC and neoplastic transformation

Cancer cells potentially arise through cancer stem cells, aberrant differentiation of tissue specific progenitor cells, transdifferentiation of differentiated cells, or a combination of these mechanisms [90–92]. All these processes involve significant gene expression changes that enable the cancer cells to adapt and survive. Reduced levels of 5hmC compared to normal tissue is a widespread phenomenon in primary cancers and has been proposed as an early diagnostic biomarker. In haematological and melanoma cancers reduced levels of 5hmC have been ascribed to loss of TET catalytic function because of mutation, cofactor availability or competitive inhibition by oncometabolites such as 2-hydroxygluterate formed by mutations in isocitrate dehydrogenase (*IDH1* and *IDH2*) enzymes. However, the majority of human cancers do not contain *TET1*, *TET2*, *TET3*, *IDH1*, or *IDH2* mutations and, still show a reduced 5hmC compared to the corresponding normal tissue. In these cases, it has been presumed that the increased proliferation rate of tumours dilutes 5hmC during DNA replication. Indeed, cells that have high Ki67, a nuclear antigen proliferation marker, stain negative for 5hmC [93]. It has also been found that within tumours, cells that stain negative for Ki67 can also be negative for 5hmC suggesting that cells with a past history of proliferation in a tumour do not regain 5hmC when cells stop cycling [93].

Another contributing factor to lower 5hmC levels in tumours may be the overall reduction in methylated DNA substrate, which is also a feature of cancer. However, the extent of loss of 5hmC does not parallel the magnitude of loss of 5mC. Moreover, the genomic regions that usually become demethylated in cancer, are within heterochromatin, where 5hmC does not normally accumulate. Within the context of global demethylation, a subset of loci gain DNA methylation at CpG islands within their promoters. These loci have been shown to be targets of polycomb repression, marked by bivalent histone modifications and low levels of expression in normal tissue and embryonic stem cells [94]. This observation of genes being silenced that are expressed at low levels in the first place, challenges previous ideas that methylation events in cancer silences tumour suppressor genes and supports a theory that methylation reduces the plasticity of gene expression [95]. In the absence of *TET* or *IDH* mutations it has been hypothesised that CpG islands susceptible to gain of 5mC in tumours are either not TET targets, or that TET target regions are protected from misdirected CpG methylation [95]. Support for this hypothesis is the observation that loci with 5hmC in normal colon tissue do not become hypermethylated in tumours [96].

**Fig. 5:**
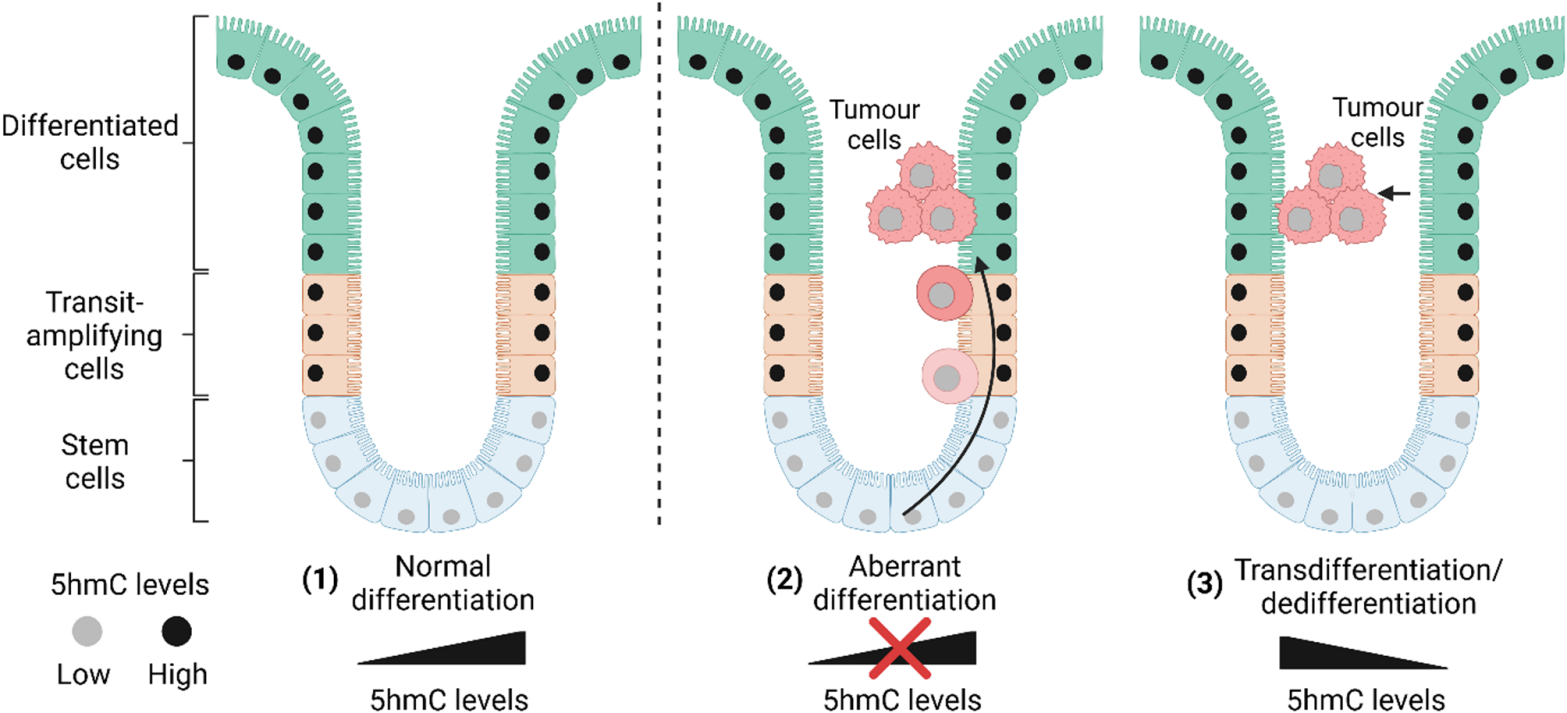
5hmC levels during neoplastic transformation. Colon villi represent a good example of committed adult progenitor cells (present in the crypts*) and epithelial differentiation along transit-amplifying cells. **(1)** Upon normal differentiation 5hmC levels that are low in the stem cells (light grey nuclei) accumulate in transit-amplifying and differentiated cells (black nuclei) **(2–3)** Neoplastic transformation in the colon is associated with reduced 5hmC in tumour cells profile of the progenitor stem cell identity. Reasons for reduced levels of 5hmC in tumours may include: **(2)** Aberrant differentiation: where the tumours initiate from the crypt but fail to accumulate 5hmC linked to a lack of 5hmC production, **(3)** Transdifferentiation: where differentiated cells acquire mutations leading to fast replicating neoplastic cells that fail to maintain 5hmC levels, and dedifferentiation: where differentiated cells revert back to a stem-like state during neoplastic transformation and lose 5hmC. *Paneth cells in the crypt are not depicted in this figure for simplicity. Created in BioRender: https://BioRender.com/f16f504

If primary cancers arise through aberrant differentiation from committed progenitor cells, as described by lineage tracing experiments by Visvader et al. [97]the lack of 5hmC could reflect the progenitor stem cell identity. In such a scenario, the progenitor cells maintain stem-like properties as cancer stem cells and do not accumulate 5hmC when they migrate or grow out of the crypt (Fig. 5 (2), middle panel). In this case, the tumour does not lose 5hmC, but rather fails to accumulate 5hmC. There are parallels between the reprogramming of somatic cells to iPSCs and dedifferentiation in cancer, such that differentiated cells may lose the expression of lineage-specific genes during oncogenic transformation and acquire a more pluripotent proliferative phenotype, suggesting that similar pathways can be associated with both the induction of pluripotency and oncogenesis [98]. If in the model as shown in (Fig. 5 (3), right-hand panel), tumours arise through transdifferentiation (or dedifferentiation) of differentiated cells, then more extensive epigenetic reprogramming including loss of 5hmC (potentially through DNA repair mechanisms or failure to maintain 5hmC) would account for the lack of 5hmC.

Recent studies have shown that compared to primary colon cancer tumours, progression to liver metastasis results in an accumulation of 5hmC, with similar locus-specific profiles as normal colon [99]. Interestingly, metastatic tumours maintain largely similar DNA methylation profiles as the primary tumour of origin [100]which fits with metastasis being a process of clonal selection [101]. Despite low levels of 5hmC in tumour tissue, there is enough 5hmC present to determine both the tissue origin and detect unique tumour and metastasis specific signatures which can be exploited diagnostically in liquid biopsies in cell free DNA detection [102]. Altogether, 5hmC is implicated in neoplastic cell conversions, and could potentially be used as an indicator for cancer.

## Conclusions and future perspectives

The past 15 years of research together highlights the essential role of 5hmC during cell conversions and creates new opportunities for its use as a cell identity marker. Recent work has already shifted towards the mapping of 5hmC across human tissue to create a DNA hydroxymethylation atlas [103, 104]as previously seen for the human proteome and DNA methylome [105, 106]. The collection of such atlases creates an elaborate reference map describing the molecular and epigenetic states of cells in normal human tissues, which can further enhance our understanding of cell identity regulation in the context of cell conversions. Combining such big data (including the incorporation of the 5hmC landscape) with machine learning approaches paves the way for enhanced predictive modelling of cell identity-specific gene expression dynamics and the identification of transcription factors that maintain such GRNs [107, 108]. For instance, recent studies have developed machine learning models based both solely on 5hmC signal and in combination with other epigenetic data, to (i) predict gene expression states across cell types, (ii) identify novel enhancer regions and their corresponding target genes, and (iii) model chromatin accessibility during cell differentiation [82, 109]. In a similar fashion, DNA sequence, histone modification and chromatin accessibility data have been integrated to create deep learning models to predict genome-wide 5hmC distribution [110]. Additionally, analogous 5hmC predictive modelling strategies have been applied to disease [111, 112]. These studies emphasize the added value of 5hmC in machine learning approaches to model cell identity dynamics.

Other cell identity-related applications of 5hmC revolve around epigenetic barriers in the framework of epigenetic memory and cell (re)programmability during cell conversions. As already briefly discussed, iPSCs often show epigenetic variation through somatic memory and aberrant reprogramming of DNA methylation and hydroxymethylation, which can be retained during subsequent conversions to other cell identities [38, 113]. Therefore, the initial cell origin used to generate iPSCs can greatly influence both iPSC reprogramming efficiency and differentiation potential [114, 115]. For example, it was shown that epigenetic memory related to DNA methylation and histone modification can predict reprogramming efficiency and differentiation capacity in an iPSC differentiation model to retinal organoids [116]. Adding 5hmC profiling to the equation could potentially open new avenues to allow for the prediction of cell programmability to target cell identities, as well as the evaluation of the degree of cell conversion following (re)programming. As presented throughout this review, 5hmC dynamics may underlie cell conversions possibly as an independent epigenetic modification and through interacting with other epigenetic mechanisms. This in turn is involved in the driving of the erasure of epigenetic memory [117]. Together, these hypotheses suggest a possible pivotal function of 5hmC in overcoming epigenetic barriers for (re)programming purposes.

This review has presented studies that may support a role of 5hmC in directing cell conversions. However, a few key questions regarding the exact working mechanisms remain. Firstly, studies have shown that 5hmC is primarily a stable epigenetic modification in mammalian cells and tissue [25] yet during cell conversions 5hmC is highly dynamic [36, 48]. The actual stability of 5hmC, whether it carries out specific biological functions as a stable epigenetic mark or only has a role as transient intermediate in DNA demethylation, during and after changing cell identity is still unclear. Secondly, although several correlations and interactions between 5hmC and other epigenetic mechanisms have been reported throughout this review, the direct and causal relationship between hydroxymethylation and other epigenetic mechanisms, including transcription factor binding, are ambiguous. Thirdly, the applications of 5hmC as an individual, standalone epigenetic mark has not yet been fully explored. Finally, many studies investigating the role of 5hmC have focused on antibody-based technologies such as hydroxymethylated DNA immunoprecipitation and immunofluorescence that lack single base resolution. However, the recent advancements in resolution and scalability of 5hmC sequencing technologies (both as a single entity and joint together with 5mC [118, 119]) and the increased availability of efficient and defined cell reprogramming models [9] creates ample opportunity to address these questions.

In conclusion, 5hmC may be an excellent epigenetic candidate for mediating cell identity changes during cell conversions. It interacts with other epigenetic mechanisms to establish an epigenetic landscape that is susceptible to reconfigurations of GRNs. Profiling of 5hmC opens up avenues for comprehensive analyses to understand and guide cell identity and epigenetic plasticity.

## Data Availability

No datasets were generated or analysed during the current study.

## Abbreviations

5cAC: 5-carboxylcytosine
5fC: 5-formylcytosine
5hmC: 5-hydroxymethylcytosine
5mC: 5-methylcytosine
ATAC: Assay for Transposase-Accessible Chromatin
BER: Base excision repair
DNMT: DNA methyltransferase
GRN: Gene regulatory network
hESC: Human embryonic stem cell
iPSC: Induced pluripotent stem cell
IDH: Isocitrate dehydrogenase
MEF: Mouse embryonic fibroblast
mESC: Mouse embryonic stem cell
OSKM: OCT4, SOX2, KLF4 and c-MYC
TDG: Thymine DNA glycosylase
TET: Ten-eleven translocation
